# Multi-Feature Fusion for Estimating Above-Ground Biomass of Potato by UAV Remote Sensing

**DOI:** 10.3390/plants13233356

**Published:** 2024-11-29

**Authors:** Guolan Xian, Jiangang Liu, Yongxin Lin, Shuang Li, Chunsong Bian

**Affiliations:** 1 State Key Laboratory of Vegetable Biobreeding, Institute of Vegetables and Flowers, Chinese Academy of Agricultural Sciences, Beijing 100081, China; 82101225079@caas.cn (G.X.);; 2 College of Agronomy and Biotechnology, Southwest University, Chongqing 400715, China

**Keywords:** UAV, above-ground biomass, multi-feature fusion, machine learning, plant phenotyping, potato

## Abstract

Timely and accurate monitoring of above-ground biomass (AGB) is of great significance for indicating crop growth status, predicting yield, and assessing carbon dynamics. Compared with the traditional time-consuming and laborious method through destructive sampling, UAV remote sensing provides a timely and efficient strategy for estimating biomass. However, the universality of remote sensing retrieval models with multi-feature fusion under different management practices and cultivars are unknown. The spectral, textural, and structural features extracted by UAV multispectral and RGB imaging, coupled with agricultural meteorological parameters, were integrated to estimate the AGB in potato during the whole growth period. Six advanced modeling algorithms, including random forest (RF), partial least squares regression (PLSR), multiple linear regression (MLR), simple linear regression (SLR), ridge regression (RR), and lasso regression (LR) models, were adopted to evaluate the ability of estimating AGB by single feature and multi-feature information fusion. The results indicate the following: (1) The newly proposed variety-dependent indicator growth process ratio (GPR) can improve the model accuracy by over 20%. (2) The fusion of vegetation indices, canopy cover, growing degree days, and GPR achieved higher accuracy to estimate AGB at all growth stages compared with single feature model. (3) RF model performed best for the estimation of AGB during the whole growth period with R^2^ 0.79 and rRMSE 0.24 ton/ha. The study demonstrated that the fusion of multi-feature coupled with the machine learning algorithm achieved the best performance for estimating potato AGB under different management practices and cultivars, which can be a potential and useful phenotyping strategy for estimating AGB at refined plot scale during the whole growth period.

## 1. Introduction

In recent years, global food security has faced challenges from multiple factors, including climate change, population growth, limitations in irrigable water resources, and instability in international markets. Food demand in the world is expected to increase by up to 70% by 2050 with increasing population. Climate change and water scarcity will lead to a reduction in potato yields [1,2,3]. Biomass is an important indicator for representing crop growth status, assessing agricultural productivity, and assessing the carbon cycle in ecosystems. Potato (*Solanum tuberosum* L.) is the third largest staple food in the world. About two-thirds of the world’s population consumes potatoes as a staple food; nearly 50% of potatoes are used as household staple food or vegetables. According to FAO statistics from 2020 to 2030, the contribution rate of potato to global food will increase from 3.74% to 6.42%, with an increase of 2.68 percentage points. Therefore, timely and accurate monitoring of its biomass is crucial for understanding the relationship between crops and the environment, food security, precision field management, crop breeding, and sustainable agricultural development. Field management and yield prediction rely on rapid, non-destructive, and accurate measurement of above-ground biomass (AGB) and its spatiotemporal dynamics [4,5,6]. However, traditional methods of measuring AGB often rely on destructive sampling techniques, such as using a spade to manually harvest crops, weigh them, and record data. These methods are time-consuming, labor-intensive, and inefficient, and they also suffer from incomplete spatial coverage [7,8]. Remote sensing offers an effective means of monitoring real-time AGB across different spatial scales throughout the various stages of crop growth [9]. Since the 1970s, non-destructive remote sensing technologies have increasingly played a significant role in spatial extrapolation and prediction of crop biomass [10]. For example, the data sources of satellite remote sensing include Landsat-8 and Sentinel-2, while the data sources for aerial unmanned aerial vehicle (UAV) remote sensing commonly include the DJI M600 and Phantom 4.

Recently, with the rapid improvement of the spatial and temporal resolution of remote sensing images, it has become an important method to construct vegetation indices using the spectral reflectance of visible light and near-infrared bands for AGB estimation. For example, the normalized difference vegetation index (NDVI) reflects crop vigor and nutritional status, the simple ratio index (SR) is sensitive to leaf area indices and canopy responses, and the renormalized difference vegetation index (RDVI) is particularly useful in areas with sparse vegetation or significant canopy variations, among others [11,12,13]. These indices are widely used for monitoring vegetation growth. Even though saturation of vegetation indices occurs to some extent as the canopy increases with the progression of the growing season, vegetation indices (VI) are effective predictors of crop AGB [14,15]. Liu et al. applied the Savitzky–Golay (SG) filter to smooth the canopy hyperspectral reflectance. They developed a model for predicting potato AGB at different growth stages by extracting deep features from hyperspectral data [16]. Zheng et al. utilized UAV digital cameras to obtain color indices, multispectral cameras to obtain red edge VIs, and color infrared cameras to obtain near-infrared vegetation indices to assess the capability of these sensors for monitoring leaf nitrogen in rice during different growth stages [17]. Liu et al. extracted optical, texture, and structural features from UAV-derived image data. The results indicate that feature fusion model performed the best in estimating AGB, with the fusion of texture and structural features showing the highest performance [18]. However, these methods only compare and analyze the monitoring effectiveness of single sensor data and spectral features at specific growth stages without considering the continuity across multiple growth stages, making it difficult to establish an estimation model applicable to the entire growth period. As crop coverage increases, the performance of AGB estimation across multiple growth sequences decreases [19]. This limitation may hinder the management and application of the AGB estimation model throughout the entire growth period of potatoes. Growth process ratio (GPR) simulates the actual developmental processes of crops, and different varieties within the same crop can have varying growth cycle durations due to differences in growth periods. Tirado et al. found that time series measurements can not only help estimate lodging and early vegetative growth rates but also aid in assessing end-of-season yield [20]. Historically, agricultural workers effectively predicted crop yields and determined appropriate harvesting times by accurately forecasting growth cycles. Nowadays, precision agriculture requires variability estimation and decision-making based on “the right time, the right quantity, and the right location” [21].

The use of single data sources for predicting AGB has its limitations, and relying solely on canopy spectral parameters poses inherent challenges. Recently, multidimensional data—including spectral information, crop canopy structure, growth temperature and agricultural meteorological parameters—have been employed to enhance AGB estimation. Texture features are based on spatial variations between image pixels and can reflect information beyond spectral features [22]. In the process of potato AGB modeling and estimation, the combination of texture features and spectral information can effectively improve the accuracy of AGB prediction at specific growth stages [23]. However, the effect of the crop growth period is not taken into account, resulting in an ineffective AGB estimation model for the whole growth period. Crop canopy coverage (CC) represents the ratio of vertical projection area of crop canopy to ground area, which can qualitatively reflect the growth trend of potato plant population structure [24,25]. Growing degree days (GDDs) is a concept that quantifies the relationship between plant phenology and temperature. It is calculated using the accumulated effective temperature values required to complete a certain growth stage, based on the maximum and minimum daily temperatures and the crop’s base temperature [26]. GDDs represent the heat accumulated during the growth period of plants and are directly related to the growth rate and growth stage of plants.

Biomass changes are largely dependent on agricultural phenological variations, and a single phenological stage cannot accurately represent the complete growth cycle. However, the Zadok scale or civilian time indicators (such as the day of the year (DOY) or days from sowing (DFS)) can be used to predict phenological stages themselves [27,28]. While Li et al. developed the CBA-Wheat model, using this method to effectively predict the total seasonal biomass of wheat’s [29] potential with this approach for predicting potato AGB, as well as the development of models that integrate multidimensional data for AGB prediction, still require further investigation.

Recent studies indicate that combining different types of features, as opposed to relying on a single sensor, has the potential to enhance model accuracy and generalizability, thereby improving crop nutrient estimation in agricultural applications [30]. In addition, the study of summer barley biomass demonstrated that combining canopy structure features with spectral characteristics significantly enhanced the model’s ability to estimate crop biomass. Meanwhile, integrating texture features with spectral characteristics has been proven to perform excellently in predicting soybean yield [31,32]. Additionally, while Liu et al. conducted extensive research on the estimation of potato AGB, there is still a lack of studies examining the impact of early, mid, and late-maturing cultivars, as well as planting density, on these estimates [18,33]. Fan et al. studied the estimation of potato yield by integrating remote sensing technology and environmental variables using a hierarchical linear model (HLM), demonstrating strong generalization ability across regions and years [34]. However, it remains unclear whether stage-specific predictions for different growth period cultivars (such as early, mid, and late-maturing) will produce good results in complex environments with varying nitrogen, potassium, and density management, and the overall predictive capability for the entire growth period has yet to be validated. Therefore, future research should consider utilizing a more diverse range of potato datasets to validate the advantages of different remote sensing features in estimating potato AGB. Current studies mainly focus on AGB estimation using single features in specific growth environments or particular cultivars [35,36], while research on estimating biomass over the entire growth period using multi-feature information fusion under various limiting factors remains relatively scarce.

The integration of multiple feature information and machine learning algorithm has good potential in crop growth monitoring; however, the universality of remote sensing retrieval models with multi-feature fusion under different nutrient supplies, plant densities, and cultivars are unknown. The study aims to (1) determine the relationship among the spectral, textural, and structural characteristics of UAV multispectral and digital data and AGB; (2) analyze the effects of two feature filter methods and six machine learning algorithms on biomass estimation; and (3) explore the potential of multi-feature fusion for estimating potato AGB in complex environments during the whole growth period.

## 2. Results

### 2.1. Correlation Analysis Between Remote Sensing Features and AGB

#### 2.1.1. Relationship Between AGB and Spectral Reflectance

**Figure 1:**
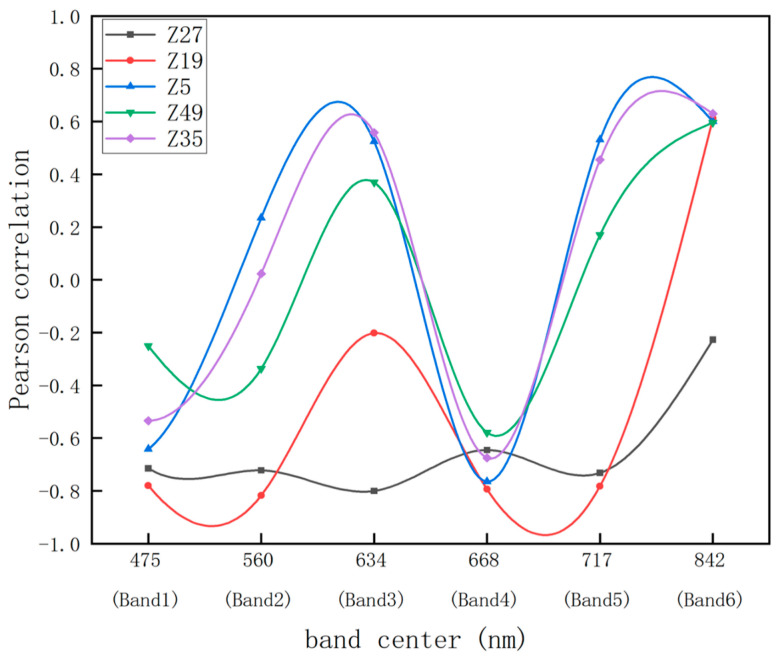
Correlation coefficients of different cultivars at different wavelengths from the band center.

Different photosynthetic pigments in plants have varying absorption preferences for different wavelengths of light. To identify the sensitive bands for different varieties, a Pearson correlation analysis was conducted. This analysis revealed the correlation of various potato varieties’ canopy original spectral bands: Band-1, Band-2, Band-3, Band-4, Band-5, and Band-6 with above-ground biomass, as shown in Figure 1. The response trend of potatoes to the original spectral bands are generally consistent, but there are differences among varieties. This is mainly due to the different growth stages of early-maturing, mid-maturing, and late-maturing varieties at the same measurement time. Based on the correlation strength represented by the absolute value of the correlation coefficient [37], Z19 shows a highly significant negative correlation with above-ground biomass (AGB) in Band-2 and a significant positive correlation in Band-6 (*p* < 0.01). The correlation coefficients are −0.817 and 0.612, respectively, indicating that Z19 has a very strong correlation with AGB in Band-2 and a strong correlation in Band-6. Similarly, Z5 shows a highly significant negative correlation with above-ground biomass (AGB) in Band-4 and a significant positive correlation in Band-6 (*p* < 0.01) with correlation coefficients of −0.765 and 0.600, respectively, indicating that Z5 is strongly correlated with AGB in both Band-4 and Band-6. Z49 also exhibits a highly significant negative correlation in Band-4 and a significant positive correlation in Band-6 (*p* < 0.01), with correlation coefficients of −0.579 and 0.596, respectively, indicating that Z49 has a moderate correlation with AGB in both bands. For Z35, there is a highly significant negative correlation in Band-4 and a significant positive correlation in Band-6 (*p* < 0.01), with correlation coefficients of −0.675 and 0.630, respectively, indicating that Z35 is strongly correlated with AGB in both Band-4 and Band-6. Z27 shows a highly significant negative correlation with above-ground biomass (AGB) in Band-3 (*p* < 0.01), with a correlation coefficient of −0.803, indicating that Z27 has a very strong correlation with AGB in Band-3. Based on the correlation analysis of multiple varieties across different bands, the sensitive bands for potatoes can be more thoroughly identified, specifically Band-4 (red band) and Band-6 (near-infrared band). Therefore, this study calculated 14 vegetation indices related to the red band and the near-infrared band.

#### 2.1.2. Relationship Between AGB and VIs

**Figure 2:**
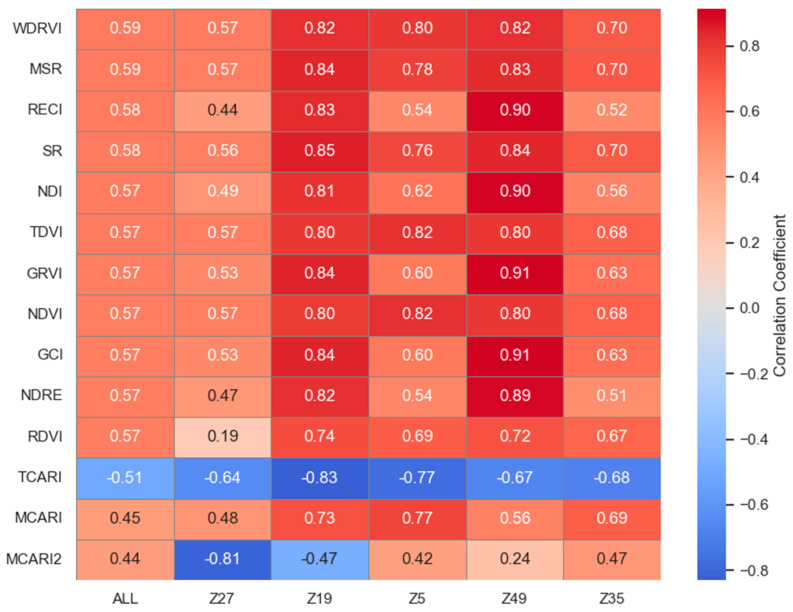
Correlation coefficients of different VIs with multiple varieties.

The Pearson correlation coefficients between the vegetation indices (VIs) extracted from multispectral images and the above-ground biomass (AGB) of potatoes throughout their growth period are shown in Figure 2. The 14 VIs calculated in this study demonstrated different relationships with AGB. In the correlation analyses between multiple varieties and VIs, the wide dynamic range vegetation index (WDRVI), which effectively distinguishes different vegetation type characteristics, shows the strongest correlation across multiple varieties (r = 0.590). This index exhibits varying performance across different varieties, with correlation coefficients of 0.575 for Z27, 0.818 for Z19, 0.803 for Z5, 0.820 for Z49, and 0.700 for Z35. Conversely, the modified chlorophyll absorption ratio index 2 (MCARI2) is sensitive to changes in chlorophyll concentration and varies significantly across different varieties. As a result, its overall representativeness in the correlation is limited. It shows a strong negative correlation with AGB in Z27 (r = −0.809), but the correlation is weaker in other varieties (r = 0.441).

**Figure 3:**
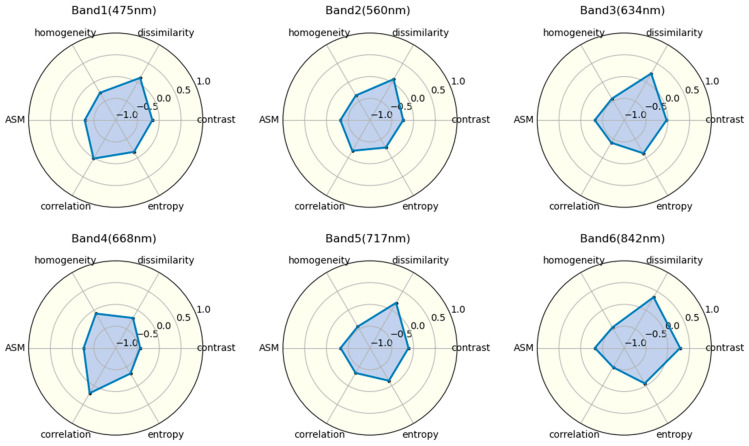
Correlation between texture features and AGB across six center bands.

Similarly, the correlation of texture features extracted from the six bands with AGB is not consistent (Figure 3). The texture feature with the strongest correlation varies by band; in Band-1, the strongest feature is ASM (r = −0.288), while the strongest features in other bands are Band-2 homogeneity (r = −0.346), Band-3 homogeneity (r = −0.430), Band-4 contrast (r = −0.438), Band-5 homogeneity (r = −0.434), and Band-6 correlation (r = −0.487). The texture features used in AGB modeling and estimation do not result in a significant underestimation of higher AGB values [18]. Therefore, we selected the three texture features with the highest “r” values using the correlation coefficient method: Band-6 correlation (r = −0.487), Band-6 homogeneity (r = −0.455), and Band-4 contrast (r = −0.438).

**Figure 4:**
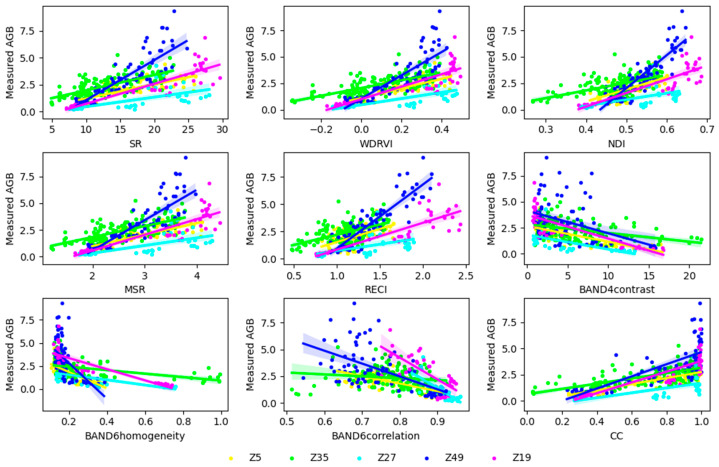
Correlation between measured AGB and modeling parameters.

Based on previous research, this study conducted a correlation analysis between 15 calculated vegetation indices (VIs) and 8 texture characteristics with biomass. The strength of the correlation between each feature and biomass was evaluated using the correlation coefficient (r), and the top five vegetation indices with the highest absolute correlation coefficients and the top three texture characteristics were selected for regression analysis [38]. Meanwhile, the correlation between CC and the measured AGB across different varieties was quantified, showing that the differences varied among the different varieties. The correlation between the measured AGB of multiple varieties and the selected features is illustrated in Figure 4.

The results in Figure 4 also indicate significant performance differences of the same VIs across different potato varieties. Specifically, among the different vegetation indices, Z49 performed the best (r ≥ 0.82), while Z27 performed the worst (r ≥ 0.44). For the other indices, Z5, Z35, and Z19 had the weakest correlations of 0.54, 0.52, and 0.81, respectively. The VIs most strongly correlated with Z35 and Z27 are WDRVI\MSR (r = 0.80), while the VI most strongly correlated with Z5, Z49, and Z19 are WDRVI (r = 0.80), RECI\NDI (r = 0.90), and SR (r = 0.85), respectively. That indicates using only one VI to estimate AGB during the growth process of multi-variety potatoes is limited.

### 2.2. Estimation of Biomass Based on Single-Type Features

#### 2.2.1. AGB Estimation Using Features Selected by Boruta

To quantify the modeling effects using only spectral feature parameters or only texture feature parameters, important vegetation indices (MCARI2 and TCARI) and texture features (BAND5 entropy, BAND6 contrast, and BAND6 ASM) were selected based on the Boruta method. This section summarizes the accuracy of estimating the AGB of potatoes using either the single data source of vegetation indices or texture features under various modeling approaches, including RF, PLSR, MLR, SLR, and Ridge and Lasso regressions. From Table 1, it can be observed that the RF model performed the best among the modeling algorithms. When using VIs to build AGB models, the R^2^ for the train and test were 0.89 and 0.65, respectively. The PLSR method followed in performance. In the texture feature modeling, the R^2^ for the training set using RF modeling was 0.82, and the R^2^ for the test set was 0.63.

#### 2.2.2. AGB Estimation Using Features Selected by Correlation Coefficient

In order to further evaluate the coupling performance of different models with various input variables, features were initially selected using the Boruta method for preliminary modeling, followed by feature selection using the correlation coefficient method. Finally, a comparative analysis of the feature selection methods was conducted based on the accuracy of the models. The modeling factors are presented in Table 2. Table 2 summarizes the accuracy of estimating the AGB of potatoes using single data sources, such as VIs or texture features, under various modeling methods, including RF, PLSR, MLR, SLR, and Ridge and Lasso regressions, based on the features selected by the correlation coefficient. As shown in the table, when utilizing VIs for modeling, the PLSR model exhibited the best performance among the tested algorithms (training: R^2^ = 0.59, rRMSE = 0.35, and MAE = 0.61; testing: R^2^ = 0.63, rRMSE = 0.33, and MAE = 0.59). In the modeling based on texture features, the RF model again achieved the highest accuracy (training: R^2^ = 0.84, rRMSE = 0.22, and MAE = 0.34; testing: R^2^ = 0.60, rRMSE = 0.34, and MAE = 0.56). Overall, among the different modeling approaches, those based on VIs yielded superior results. The fitted MAE for the prediction model of AGB content in the test set using VIs ranged from 0.59 to 0.78 ton·ha^−1^, with rRMSE values spanning from 0.33 to 0.43 ton·ha^−1^. In contrast, the fitted MAE for the models utilizing texture features ranged from 0.56 to 0.99 ton·ha^−1^ in the test set, with rRMSE values between 0.34 to 0.56 ton·ha^−1^.

#### 2.2.3. Analysis of Optimal Results for Single Feature Modeling

In single feature modeling (Table 1 and Table 2), when comparing different modeling features, the model based on VIs demonstrated better performance. The performance of modeling using multispectral texture feature information was slightly lower than that using VIs. Among the various modeling feature methods, the models obtained using the RF method consistently showed higher accuracy than those obtained using other methods. The results indicate that in single feature modeling, the RF model built using VIs features achieved the highest accuracy.

### 2.3. Biomass Estimation Using Multi-Feature Fusion

#### 2.3.1. AGB Estimation Based on Boruta-Selected Features

To explore the impact of multi-feature data fusion on the accuracy of potato AGB prediction models, various types of features from UAV data, GDD, and GPR were used. Six inversion methods consistent with single feature-type modeling were utilized for AGB estimation, and optimal fused feature combinations under the same modeling method were organized, as shown in Table 3. Regardless of the modeling method used, the prediction models constructed using a fusion of vegetation index, texture features, canopy coverage, growing degree days, or growth sequences performed better with higher estimation accuracy compared to models that only used a single feature within the same machine learning method. Among them, the RF model achieved the highest accuracy (train: R^2^ = 0.88~0.90, rRMSE = 0.16~0.18, and MAE = 0.25~0.28; test: R^2^ = 0.73~0.78, rRMSE = 0.24~0.27, and MAE = 0.38~0.42). Notably, in the RF model, the combination of VIs + GDD + CC + GPR performed the best among all methods and combinations (train: R^2^ = 0.90, rRMSE = 0.16, and MAE = 0.25; test: R^2^ = 0.78, rRMSE = 0.24, and MAE = 0.38). Additionally, the optimal combinations of modeling features were inconsistent across different modeling methods. In the MLR model, the best feature combination was VIs + GPR. For the Lasso, PLSR, Ridge, and SLR models, the optimal feature combinations were VIs + GDD + CC + GPR, VIs + Texture, Texture + GDD + CC + GPR, and VIs + Texture + GDD + CC + GPR, respectively.

#### 2.3.2. AGB Estimation Based on Correlation Coefficient Filtered Features

To explore the impact of different feature selection methods on the estimation of potato AGB through multi-feature fusion, features selected by the correlation coefficient were fused with other features to conduct multi-feature fusion modeling, using the same modeling methods as described earlier. The results are summarized in Table 4, highlighting the three optimal feature combinations under the same modeling method. The data indicate that this approach is consistent with conclusions from multi-feature modeling using the Boruta method. Regardless of the modeling method used, predictive models constructed by fusing the VIs, texture features, CC, GDD, or GPR exhibited higher estimation accuracy compared to models that utilized only a single feature type. Notably, the RF model achieved the highest accuracy (train: R^2^ = 0.79~0.91, rRMSE = 0.16~0.24, and MAE = 0.25~0.39; test: R^2^ = 0.76~0.79, rRMSE = 0.28~0.29, and MAE = 0.38~0.46). For the MLR model, the best feature combination was VIs + CC. In the Lasso, SLR, PLSR, and Ridge models, the optimal combinations were the following: VIs + GDD + CC + GPR, VIs + texture + GDD, VIs + texture + GDD + CC, and VIs + GDD + CC, respectively. In these six modeling methods, the optimal feature combinations were all composed of the VIs combined with another feature, thereby becoming the optimal model under each machine learning technique. The results indicate that when considering multi-feature inputs and different machine learning algorithms, the combination of VIs with other features shows significantly higher overall accuracy than other feature fusions. This suggests that VIs can effectively predict and enhance the spectral characteristics of vegetation, while its strong regional specificity and saturation issues can also be improved through fusion with other features.

#### 2.3.3. Analysis of Optimal Results for Multi-Feature Modeling

It is worth mentioning that in the RF model (Table 3 and Table 4), the combination of VIs and GPR performed best among all models based on the correlation coefficient filtered feature combinations (train: R^2^ = 0.89, rRMSE = 0.17, and MAE = 0.29; test: R^2^ = 0.79, rRMSE = 0.29, and MAE = 0.46). Moreover, the optimal combinations of modeling features varied across different modeling methods based on correlation coefficient filtering. Among the best feature combinations of the six methods, five of the optimal feature combinations included the VIs or GPR, highlighting the critical importance of VIs and GPR in improving the accuracy of biomass estimation models. Additionally, considering the modeling algorithms, the performance of the RF model was generally superior to that of other algorithms under different feature combination inputs, indicating that the RF model exhibited better stability. In conclusion, the fusion of multi-feature information significantly enhanced the estimation performance of the models.

### 2.4. The Impact of Different Feature Selection Methods on AGB Estimation

**Figure 5:**
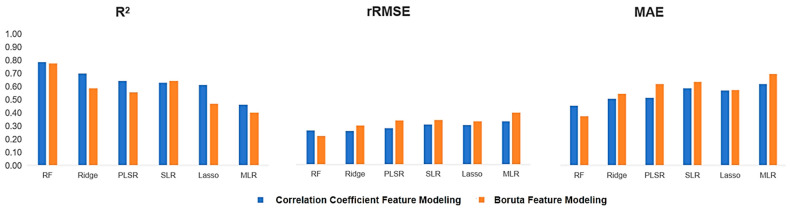
Comparison of modeling accuracy of machine learning under two feature selection methods.

Feature selection involves identifying and retaining the most representative and significant features from the original data while removing irrelevant or redundant ones. The main objectives are to enhance model accuracy and precision, reduce the risk of over fitting, lower the number of features to simplify the model, shorten training time, save computational resources, and improve interpretability and visualization. In this study, we employed two feature selection methods and used the same machine learning techniques and feature combinations for analysis. As shown in Figure 5, feature selection led to a significant decrease in model performance for the Ridge, PLSR, Lasso, and MLR methods, while improving the performance of other models, such as SLR. Among the various machine learning algorithms and feature combinations assessed, the RF method, due to its advantage in handling multi-source data and its superior estimation accuracy and robustness, provided the most accurate results in biomass estimation. This was followed by the Ridge method, while the MLR method exhibited the largest error compared to actual measurements.

**Figure 6:**
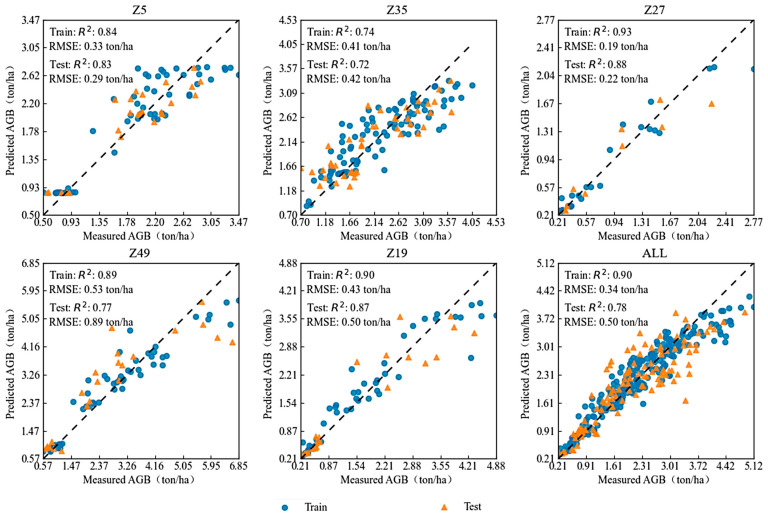
Multi-variety modeling results based on Boruta selection of VIs + GDD + CC + GPR fusion.

**Figure 7:**
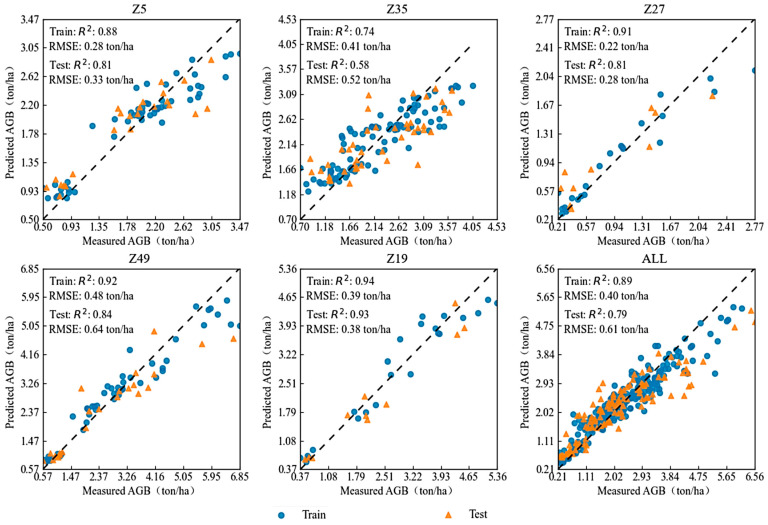
Multi-variety modeling results based on correlation coefficient selection of VIs + GPR fusion.

Therefore, the RF method was used for modeling validation of single varieties over the entire growth period, as shown in Figure 6 and Figure 7. Different feature selection methods affected the optimal feature combinations for modeling. Under the Boruta method, the combination of VIs + GDD + CC + GPR achieved the highest modeling accuracy, while under the correlation analysis method, the combination of VIs + GPR provided the best performance. GPR offered valuable information related to expected AGB at any point during the growth period, and its fusion with remote sensing indices (VIs) effectively predicted the AGB for the early-, mid-, and late-maturing varieties involved in this study. In modeling, a higher R^2^ and lower RMSE on the test set indicate better prediction accuracy of AGB and smaller errors compared to actual measurements. When modeling the same dataset, the differences between methods are mainly due to differences in model structures. Under the Boruta method, when the combination of VIs + GDD + CC + GPR was used, the model validation R^2^ values for Z5, Z35, Z27, Z49, and Z19 were 0.83, 0.72, 0.88, 0.77, and 0.87, respectively, with RMSE values of 0.29, 0.42, 0.22, 0.89, and 0.50 ton/ha. In the correlation analysis method, when VIs + GPR was used, the model validation R^2^ values for the same varieties were 0.81, 0.58, 0.81, 0.84, and 0.93, with RMSE values of 0.33, 0.52, 0.28, 0.64, and 0.38 ton/ha, respectively. In summary, under the Boruta feature selection method, the validation R^2^ for AGB predictions of all varieties was above 0.72. However, under the correlation feature selection method, the validation accuracy for Z35 was lower (R^2^ = 0.58), while the other varieties achieved validation R^2^ values above 0.81.

## 3. Discussion

### 3.1. Multi-Feature Fusion for Crop AGB Prediction

Multi-feature fusion, as an interdisciplinary field based on modern information technology, has achieved significant research value and broad application prospects across various domains. The shift from single feature to multi-feature integration plays a crucial role in agriculture, impacting crop growth monitoring, yield prediction, disaster monitoring, and precision management. This integration helps improve the efficiency, reliability, and service levels of agricultural operations, representing a typical exploration towards intelligent, technological, and modernized agricultural production [39]. VIs are effective indicators for estimating potato above-ground biomass (AGB). However, a common limitation in studies using VI data for AGB modeling is that these models are often developed and tested at specific time points, typically within fixed nutritional growth sequences [40]. In precision agriculture, the fusion of multiple features provides a more comprehensive view of crop conditions. VIs reflects the growth and health of potatoes, while GDD indicates the growth progress and developmental stage. CC helps assess the vigor of potato plants, and GPR offers precise information about the variety’s growth period. By integrating these diverse data points, a clearer understanding of the overall state of the potato field is gained, enabling more informed and accurate decision-making. Relying on a single feature may have limitations, but combining multiple features allows them to complement each other, enhancing the precision of decisions [34]. By combining different features, diverse information dimensions can be provided. When the vegetation index saturates, introducing GDD and GPR, which can continuously provide information about crop growth progress, might help alleviate the saturation problem. This can be further explored in future research. The results of this study confirm that it is impractical to construct a universal model for AGB estimation across multiple varieties using only VI. Therefore, when building AGB estimation models for various varieties over their entire growth periods, it is essential to integrate remote sensing data, meteorological information, and phenological stage data. The findings indicate that potato AGB estimation models based on fused GPR outperform those using single remote sensing features in multi-variety predictions. This improvement may be attributed to the rich data absorption from diverse dimensions through multi-feature information fusion, aligning with the findings of Xu et al. [41]. Consequently, considering feature fusion methods is crucial for drone-based remote sensing monitoring of potato AGB and can also provide insights for research on other crops like wheat and rapeseed.

### 3.2. Evaluation of GPR Application in AGB Prediction

Potatoes are crops that use underground tubers as their economic organs, with a growth cycle ranging from 70 to 115 days. There are significant differences in above-ground biomass (AGB) across varieties at different growth stages. Spectral characteristics serve as the physical basis for distinguishing crop types, and due to the typical vegetation spectral features of crops, the visible to near-infrared range exhibits a prominent “double peak and double valley” pattern [42], making it rich in data characteristics. Different potato varieties or stages of growth exhibit distinct spectral responses, so to better estimate the above-ground biomass of different potato varieties using spectral reflectance, it is essential to account for the differences in growth periods among them.

The varieties used in this study include the early-maturing Zhongshu 5 (75 days) and Zhongshu 35 (75 days), mid-maturing Zhongshu 27 (95 days), and late-maturing Zhongshu 19 (105 days) and Zhongshu 49 (110 days), with a maximum growth period difference of 35 days. GPR is the ratio of sampling time to the growth cycle of a variety, and it can quantify the key stages of the growth process, helping to distinguish the growth statuses of different varieties. When constructing the estimation model, real-time growth periods of different varieties were quantified using GPR and integrated with other features for estimation. Different varieties have distinct growth cycles, including differences in sowing time, emergence time, growth rate, and maturity time. This may lead to different varieties of potatoes being at different growth stages at the same sampling time, increasing the variability of the data. However, GPR can quantify these differences in growth cycles. After quantifying the growth cycles of different varieties, integrating other features into the modeling process can minimize prediction errors. The results indicate that the inclusion of GPR significantly improved model accuracy. This study tested a limited number of varieties; further research is needed to test more varieties, develop methods for quantifying growth stages, and explore other modeling forms related to AGB. There is potential for further investigation into the utility of the proposed RF model combined with GPR for predicting yield and crop carbon dynamics. In essence, growth stages are always ordinal values within the growth process, rather than continuous values. Each discrete value is associated with a phenological stage, and growth stage information can be easily confirmed through planting time and the characteristics of the variety’s growth period. The source of this information is commonly collected agricultural data during the growing season. Previous research on wheat biomass prediction has quantified growth stages and combined them with remote sensing data and phenological information to develop algorithms for predicting biomass across different growth periods [43], which have proven very effective in segmented models.

Studies on corn have shown that the assessment of crop conditions aligned with growth stages is consistent with National Agricultural Statistics Service (NASS) reports, and the introduction of growth degree units provides phenological information for corn crops [43]. This indicates that it can help improve predictions for crop yield, biomass, and Leaf Area Index (LAI) by effectively quantifying the relationship between crop growth changes and vegetation indices. Perhaps a reasonable classification of growth stages is a key step in guiding the automatic management of field fertilizers or irrigation, and it may also be a breakthrough point for developing more generalized models in remote sensing predictions. The promotion of potato varieties varies across different cultivation areas due to climatic and geographical reasons, and the growth periods of different varieties also differ. This is one reason why previous research models could not be universally applied [44]. Similarly, in this study, the introduction of GPR during the biomass estimation process for different potato varieties throughout their growth periods yielded ideal results within the RF model. The inclusion of GPR reduced the variety effects, enhancing the accuracy of AGB predictions using remote sensing images, thereby improving the model’s generalization capability.

### 3.3. Impact of Different Modeling Algorithms and Feature Selection Methods on AGB Prediction

Feature selection is a crucial preprocessing step before conducting machine learning. As datasets become larger and more complex, the number of features often increases dramatically. However, not all features contribute to the model’s performance; some may be redundant, noisy, or unrelated to the target variable. Therefore, feature selection is an indispensable part of the machine learning workflow. This study employed two feature selection methods: correlation analysis in the filter-based approach and the Boruta machine learning algorithm in the embedded approach. The former is computationally efficient and can quickly eliminate a large number of irrelevant features, but it may overlook interactions between features, leading to the removal of important ones [38]. The latter is also computationally efficient and can consider interactions between features, but it relies on specific machine learning algorithms, and different algorithms may provide varying assessments of feature importance [45]. Based on the results, the RF model’s inherent advantages might explain why there was no difference between the two feature selection methods, while other models exhibited varying results. This result is consistent with the research of Li et al., where the RF algorithm demonstrated superior performance in AGB estimation. This is because the RF has strong overfitting resistance, effectively handles high-dimensional data and nonlinear relationships, and is robust to noise and outliers [40,46]. The features provided by the same selection method are limited, so future research could integrate feature selection with data fusion to reduce redundancy and increase data diversity and model stability.

Combining machine learning algorithms with remote sensing data has been widely applied in crop monitoring and other fields due to its efficiency in handling complex, high-dimensional data, thus improving model accuracy. Therefore, this study constructed models using MLR, SLR, Ridge, Lasso, PLSR, and RF algorithms. To explore the relationship between potato AGB and VIs, texture, CC, GDD, and GS, various combinations of features were tested, revealing that the RF model achieved the highest accuracy when using VIs + CC + GDD + GS as input variables. Furthermore, to fully consider the redundancy and collinearity between feature variables, input variables were reselected based on Pearson correlation analysis. The results showed that using VIs + GS as input variables yielded the highest accuracy for the RF model. The RF model demonstrated an R^2^ of 0.79, a relative root mean square error (rRMSE) of 0.29 ton·ha⁻^1^, and a mean absolute error (MAE) of 0.46 ton·ha⁻^1^, consistent with findings by Han et al. [47] in corn AGB monitoring. The RF model, utilizing a decision tree system, effectively handled large datasets, accurately assessed feature importance, mitigated overfitting, and exhibited robustness against outliers, making it a suitable solution for inversion problems [48,49]. Thus, the integration of feature variables with regression algorithms through the RF modeling method is significant for remote sensing monitoring of potato biomass based on drone imagery. By accurately estimating above-ground biomass, it is possible to better understand crop growth conditions and needs, optimize resource management, support decision-making, and improve agricultural productivity and economic efficiency.

Biomass estimation provides essential foundational data for yield prediction, resource management, and agronomic practices. By accurately assessing the growth status and health of crops, it effectively guides the rational allocation and utilization of resources, while also helping to formulate suitable agronomic strategies. This optimization enhances crop growth and ultimately improves yield and agricultural production efficiency. Additionally, the research should extend over the entire growth cycle to comprehensively monitor potato growth dynamics and provide more extensive data support for agricultural production management. Furthermore, considering the growth differences of crops in various regions and climatic conditions, future studies should include cross-regional validation and model optimization to ensure the model’s universality and reliability.

## 4. Materials and Methods

### 4.1. Test Site

**Figure 8:**
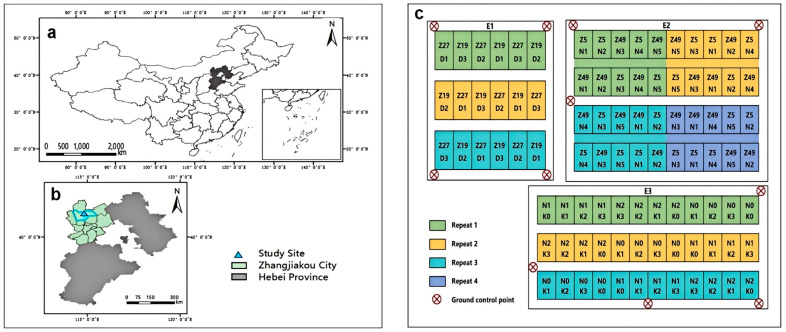
Location of the study site (**a**,**b**) and experimental design of the study (**c**).

The study area is situated in the Potato Research Base of the Institute of Vegetables and Flowers, Chinese Academy of Agricultural Sciences, located in the Chabei Management Area, Zhangjiakou, Hebei Province, China (115°03′ E, 41°28′ N, altitude 1390 m) (Figure 8a,b). This area is in the temperate arid and semi-arid zones of the East Asian continental monsoon climate, characterized by concurrent rainfall and heat, concentrated precipitation, significant diurnal temperature variation, and abundant sunlight. The average annual temperature is 2.9 °C, with an average annual effective accumulated temperature of 2029.3 °C for temperatures ≥ 10 °C. The average frost-free period is 106 days, the average annual precipitation is 381.4 mm, and the average annual sunshine amount is 2931.7 h. The plants were grown in a sandy loam field with chestnut-calcareous soil, characterized by a pH of 8.2, organic matter concentration of 25.2 g kg^−1^, soluble salts of 0.0647%, total N of 0.166%, hydrolyzable N of 71.5 mg kg^−1^, Olsen-P of 11.8 mg kg^−1^, and readily available K of 118 mg kg^−1^.

### 4.2. Experimental Design

In order to evaluate the applicability of the model in the multiple cultivars studied, three field experiments considering different cultivars (Figure 8c), plant density, nitrogen and potassium application rates were conducted.

Experiment 1 (E1) was designed to explore the interaction between cultivars and density, using the cultivars Zhongshu 27 (Z27) and Zhongshu 19 (Z19). Three density levels were established: 52,500 (T1), 60,000 (T2), and 7500 (T3) plants/ha. The experiment employed a randomized block design with three replications, and the study area contained 18 experimental plots.

Experiment 2 (E2) was designed to explore the interaction between cultivars and nitrogen dosage, using the cultivars Zhongshu 5 (Z5) and Zhongshu 49 (Z49). Five nitrogen levels were established: 0 (N0), 50 (N1), 100 (N2), 250 (N3), and 400 (N4) kg/ha. The experiment utilized a randomized block design with four replications, and the study area contained 40 experimental plots.

Experiment 3 (E3) was designed to explore the interaction between cultivars and the combination of nitrogen and potassium. The experiment utilized the cultivar Zhongshu 35 (Z35) and included three nitrogen levels, 0, 120, and 240 kg/ha, as well as four potassium levels, 0, 120, 240, and 360 kg/ha. The experiment employed a randomized block design with three replications, and the study area contained 36 experimental plots.

To ensure geometric correction, data registration, precision enhancement, and multi-source data fusion, 11 ground control points (GCPs) were evenly distributed (Figure 8c), and their positions were measured using real-time kinematic GPS (RTK-GPS).

### 4.3. Data Collection

#### 4.3.1. Ground Data Acquisition and Processing

The biomass data for potatoes were collected during four key growth stages: tuber formation stage (S1), tuber expansion stage (S2), starch accumulation stage (S3), and mature harvest stage (S4) (Table 5). The sampling method involved selecting six representative plants from each plot that effectively reflected the growth status. For the fresh biomass, the samples were cleaned, the roots were clipped, and then the weight of the stems and leaves was measured. The fresh weights of the plant stems and leaves were summed to obtain the total fresh weight of the sample. Finally, this fresh weight, along with the planting density, was used to calculate the potato above-ground biomass (AGB) for each plot [24]. The formula for calculating AGB per hectare is as follows:

AGB = AGB_ave_ × n (1)

AGB_ave_ represents the average biomass of potato plant samples, while n denotes the number of potato plants per hectare estimated based on the plot density. These conversions are essential due to the differences in density levels between the density experiment and the other two experiments.

#### 4.3.2. Meteorological Data Acquisition and Collection

The cultivars used in this study include early-, mid-, and late-maturing varieties. Due to the differing growth periods of these varieties, a single phenological stage cannot accurately describe the complete growth cycle. Therefore, we propose growth period ratio (GPR) as the proportion of growing days after planting to the total phenology days to represent the growth process of different cultivars. Specific values can be calculated later In this study, samples were collected at different times for four growth stages across five varieties to complete the quantification of the growth process. The formula for calculating GPR is as follows:

(2) $$\mathrm{GDDs}=\sum \left[(T_{\max}+T_{\min})/2-T_{b}\right]$$

T_max_ represents the maximum temperature for one day, T_min_ represents the minimum temperature for one day, and T_b_ is the base temperature for crop development.

#### 4.3.3. UAV Data Acquisition and Processing

The UAV sensors utilized in this study included multispectral and RGB. The spectral images were captured using the RededgeP multispectral lens as the remote sensing platform. The band parameter information for the built-in multispectral camera is presented in Table 6. Multispectral and RGB images were obtained for the potato tuber formation stage, tuber expansion stage, starch accumulation stage, and mature harvest stage (Table 5). The flight was conducted between 11:00 and 13:00, when sunlight intensity was stable, and the sky was clear with no wind or clouds. The flight altitude was set at 20 m, with a flight line overlap of 85%. After each flight, the images were checked for any missing data. The DJI Inspire 2 platform is equipped with RGB and multispectral sensors to collect images, with sensor information provided in Table 6. The band information used in the study corresponds to the factory-set bands of the sensors, and the subsequent data extraction and preprocessing steps are consistent with previous studies [47]. In this study, Agisoft Metashape 2.02 was used to stitch the images into orthophotos, followed by geometric and radiometric corrections using the built-in program. Subsequently, vector plots of the polygons were created in ArcGIS, and Python (version 3.10.9) software was employed to extract the average spectra for each polygon, yielding the canopy spectral reflectance for each area.

### 4.4. Analysis of UAV Remote Sensing Image Information

#### 4.4.1. Extracting Vegetation Indices

Multispectral cameras used in agriculture operate in the visible and infrared regions of the electromagnetic spectrum to precisely capture spectral measurements sensitive to plant canopy and soil reflectance, enabling effective monitoring of vegetation health and crop growth status. Vegetation reflects more in the green region compared to the red, and green band reflectance also is utilized in vegetation index calculations in the Table 7. The surfaces of plant leaves exhibit strong absorption characteristics in the visible red spectrum and strong reflection characteristics in the near-infrared spectrum. In this study, 14 vegetation indices were utilized, with specific names, calculation formulas, and references provided in Table 7.

#### 4.4.2. Extracting Texture Features

Using the gray-level co-occurrence matrix (GLCM), texture features were extracted from the R, G, B, red edge, and NIR bands of MS, as well as from the RGB grayscale image [21]. A total of eight GLCM-based texture features were derived, including mean (ME), variance (VA), dissimilarity (DI), contrast (CON), uniformity (HO), second moment (SE), correlation (COR), and entropy (EN) [58].

#### 4.4.3. Extraction of Canopy Structure Information

Canopy cover (CC) has a direct impact on plant development and crop growth, serving as an indicator of growth density and an important biophysical metric for assessing the potential for crop radiation interception [59]. Various methods exist for extracting canopy cover, including the threshold binary method, index time series method, and sample statistical method [60,61]. The RGB camera has a higher resolution, allowing it to provide clearer details of plant images.

In this study, we utilized UAV RGB imagery along with ArcGIS (10.8, ESRI, Redlands, CA) to extract canopy cover for potatoes at different growth stages (tuber formation stage, tuber expansion stage, starch accumulation stage, and mature harvest stage). The process involved first calculating the vegetation index for each growth stage, then applying the bimodal method to differentiate between vegetation and soil mixed pixels, and finally computing the ratio of vegetation pixel count to total pixel count within each sample plot to determine the extracted CC value.

### 4.5. Quantification of Growth Process Ratio

The cultivars used in this study include early-, mid-, and late-maturing varieties. Due to the differing growth periods of these varieties, a single phenological stage cannot accurately describe the complete growth cycle. Therefore, we propose using the growth days/growth period ratio (GPR) to represent the growth process. Specific values can be found in Table 8. In this study, samples were collected at different times for four growth stages across five varieties to complete the quantification of the growth process. The formula for calculating GPR is as follows:

(3) $$GPR=\frac{T_{1}}{T_{O}}$$

T_1_ represents the number of days after emergence, and T_O_ represents the total growth period in days for this variety.

### 4.6. Modeling Method

#### 4.6.1. Feature Selection Methods

(1) Feature selection based on Boruta

Having too many variables can lead to overfitting and slow down computation, hindering model generalization. Boruta is a heuristic algorithm based on random forests that constructs shadow features, allowing machine learning algorithms like random forests to directly select the optimal feature set [62]. Therefore, using Boruta for feature selection helps eliminate redundant variables, enhancing accuracy and positively influencing model precision with relevant variables. To avoid issues of multicollinearity among input variables that could distort model estimates, features with severe collinearity were excluded by calculating the variance inflation factor (VIF). The remaining features were used as independent variables, with biomass as the dependent variable, and six machine learning algorithms were employed to build estimation models for the above-ground biomass of potatoes across multiple varieties at whole growth stages.

VIF = 1/(1 − R^2^) (4)

R^2^ is the coefficient of determination, representing the extent to which a particular independent variable explains the variation in dependent variables in regression analysis.

(2) Feature selection based on Pearson correlation coefficient (r)

The strength of the correlation between each feature and biomass was evaluated using the Pearson correlation coefficient (r). According to previous studies, the top five vegetation indices with the highest absolute correlation coefficients and the top three texture characteristics were selected for regression analysis [38]. Meanwhile, the correlation between crop growth density (CC) and measured above-ground biomass (AGB) for different varieties was quantified, revealing variation among the different varieties. The correlations between the measured AGB and the selected features are shown in Figure 3. Similarly, the feature parameters selected using the correlation coefficient method were used to construct estimation models for the above-ground biomass of potatoes across multiple varieties at whole growth stages using six machine learning algorithms. Finally, a comparison was made between the two feature selection methods regarding their application and evaluation across different machine learning models.

#### 4.6.2. Model Algorithm

Random forest (RF) is a machine learning method that creates training sets by repeatedly sampling through bootstrap sampling. It uses an ensemble of decision trees to predict the dependent variable. RF is a parallel learner based on decision trees, which helps to reduce overfitting to some extent [63]. Partial least squares regression (PLSR) integrates multiple linear regression, canonical correlation analysis, and principal component analysis, providing a method for multivariate linear regression modeling that can eliminate correlations among independent variables and estimate the dependent variable with less data [64]. Ridge regression (RR) is a biased estimating regression method used for analyzing collinear data and is an improvement over the least squares estimation method. It sacrifices the unbiased advantage of the least squares method in exchange for the stability of regression coefficients, at the cost of losing some information and reducing fitting accuracy [65]. Due to the simplicity, interpretability, and ease of modeling of linear regression models, this study also employed multiple linear regression (MLR), simple linear regression (SLR), and lasso regression (LR) to model and estimate the above-ground biomass (AGB) of multiple potato varieties. RF has high accuracy, especially on complex datasets. PLSR performs well in handling high-dimensional data and nonlinear relationships but may not be as effective as RF. The accuracy of MLR and SLR depends on the linearity of the data; if the data meet the linearity assumption, good results can be achieved. Ridge and Lasso can improve the model’s accuracy when dealing with multicollinearity issues, ultimately selecting the optimal results.

### 4.7. Evaluation Indicators

In this study, two-thirds of the data samples were selected for modeling, while one-third of the samples were used as a validation set to construct the estimation model for the above-ground biomass of potatoes. The performance of the model was evaluated using the validation set’s R^2^, mean absolute error (MAE), root mean square error (RMSE), and root relative mean squared error (rRMSE). Smaller values of MAE, RMSE, and rRMSE indicate better predictive performance of the model. The expressions are as follows:

$$R^{2}=1-\frac{\sum_{i-1}^{n}{\left({\hat{y}}_{i}-y_{i}\right)}^{2}}{\sum_{i-1}^{n}{\left(y_{i}-\bar{y}\right)}^{2}}$$

$$RMSE=\sqrt{\frac{\sum_{i-1}^{n}{\left(y_{i}-{\hat{y}}_{i}\right)}^{2}}{n}}$$

$$MAE=\frac{1}{n}\sum_{i=n}^{n}\left|y_{i}-{\hat{y}}_{i}\right|$$

$$\mathrm{rRMSE}=\frac{\sqrt{\sum_{i-1}^{n}{\left(y_{i}-{\hat{y}}_{i}\right)}^{2}/n}}{\bar{y}}$$

n represents the total number of samples in the test set. y_i_ and ${\hat{y}}_{i}$ refer to the measured and predicted values of biomass, respectively. $\bar{y}$ represents the average of the measured biomass values.

## 5. Conclusions

The spectral, textural, and structural features extracted by UAV multispectral and RGB imaging, coupled with agricultural meteorological parameters, were integrated to estimate the AGB in potato during the whole growth period. The following conclusions can be drawn from this study:

1. The relationship between AGB and spectral features shows significant differences among different potato varieties. Compared to single feature modeling, integrating VIs, CC, GDD, and GPR results in a higher estimation accuracy of AGB throughout the entire growth period of potatoes.
2. The newly proposed variety-dependent indicator, growth process ratio (GPR), can improve model accuracy by over 20%.
3. The RF model using the Boruta feature selection method performed best for the estimation of AGB during the whole growth period, with R^2^ 0.79 and rRMSE 0.24 ton/ha. This model shows great potential for estimating AGB throughout the entire growth period of multiple potato varieties.

This study integrated diverse data to estimate the AGB of potatoes throughout the entire growth period across multiple varieties, different nitrogen and potassium management practices, and different planting densities. The results demonstrated the potential of the RF model to predict the above-ground biomass of potatoes across different varieties using remote sensing, meteorological, and agronomic data, providing a potential strategy for high-throughput crop phenotyping.

## Figures and Tables

**Table 1:** Modeling results of single feature types based on Boruta filtering for calibration and test datasets.

Features	Models	Model Index	Train			Test		
			R^2^	rRMSE	MAE	R^2^	rRMSE	MAE
VIs	Lasso	MCARI2 TCARI	0.05	0.54	0.96	0.06	0.52	0.89
	MLR		0.34	0.44	0.75	0.24	0.49	0.85
	Ridge		0.39	0.42	0.70	0.32	0.48	0.80
	SLR		0.24	0.54	0.90	0.40	0.44	0.77
	PLSR		0.44	0.39	0.66	0.43	0.45	0.68
	RF		0.89	0.17	0.27	0.65	0.30	0.47
Texture	MLR	BAND5entropy BAND6contrast BAND6ASM	0.08	0.52	0.92	0.05	0.55	0.97
	PLSR		0.20	0.49	0.83	0.09	0.51	0.93
	Lasso		0.08	0.51	0.88	0.09	0.56	1.01
	SLR		0.15	0.55	0.97	0.14	0.57	1.01
	Ridge		0.15	0.51	0.84	0.21	0.48	0.89
	RF		0.82	0.21	0.35	0.63	0.32	0.49

**Table 2:** Modeling results of single feature types based on correlation coefficient filtering.

Features	Models	Model Index	Train			Test		
			R^2^	rRMSE	MAE	R^2^	rRMSE	MAE
VIs	MLR	GRNDVI SR WDRVI MSR RECI	0.39	0.44	0.81	0.25	0.43	0.78
	SLR		0.32	0.46	0.86	0.39	0.41	0.71
	Lasso		0.53	0.36	0.65	0.57	0.39	0.66
	RF		0.88	0.19	0.31	0.58	0.35	0.59
	Ridge		0.60	0.35	0.59	0.60	0.33	0.61
	PLSR		0.59	0.35	0.61	0.63	0.33	0.59
Texture	MLR	Band6correlation Band6homogeneity Band4contrast	0.11	0.54	0.94	0.03	0.49	0.85
	PLSR		0.19	0.48	0.83	0.06	0.56	0.99
	Lasso		0.17	0.50	0.87	0.12	0.51	0.90
	SLR		0.27	0.48	0.85	0.21	0.46	0.82
	Ridge		0.35	0.44	0.75	0.30	0.45	0.75
	RF		0.71	0.23	0.38	0.60	0.35	0.56

**Table 3:** Modeling results of multiple features based on Boruta filtering.

Models	Optimal Feature Fusion	Train			Test		
		R^2^	rRMSE	MAE	R^2^	rRMSE	MAE
MLR	VIS + GS	0.48	0.39	0.65	0.40	0.44	0.71
PLSR	VIS + Texture	0.39	0.42	0.71	0.56	0.37	0.63
RF	VIS + GDD + CC + GS	0.90	0.16	0.25	0.78	0.24	0.38
Lasso	VIS + GDD + CC + GS	0.39	0.44	0.75	0.47	0.37	0.59
Ridge	Texture + GDD + CC + GS	0.61	0.35	0.59	0.59	0.33	0.56
SLR	VIS + Texture + GDD + CC + GS	0.60	0.38	0.64	0.64	0.38	0.65

**Table 4:** Modeling results of multiple features based on correlation coefficient filtering.

Models	Optimal Feature Fusion	Train			Test		
		R^2^	rRMSE	MAE	R^2^	rRMSE	MAE
MLR	VIS + CC	0.52	0.39	0.67	0.46	0.36	0.63
RF	VIS + GS	0.89	0.17	0.29	0.79	0.29	0.46
Ridge	VIS + GDD + CC	0.66	0.33	0.55	0.70	0.28	0.52
SLR	VIS + Texture + GDD	0.54	0.37	0.63	0.63	0.33	0.60
Lasso	VIS + GDD + CC + GS	0.57	0.36	0.62	0.61	0.33	0.58
PLSR	VIS + Texture + GDD + CC	0.63	0.34	0.57	0.64	0.31	0.52

**Table 5:** Information on the different growth stages of varieties, nitrogen, and potassium trials data collection.

Experiment	Date of UAV Flights	Date of Field Sampling	Samples	Growth Stage
1	4 July 2023	4 July 2023	18	Tuber formation
	17 July 2023	17 July 2023	18	Tuber expansion
	3 August 2023	3 August 2023	18	Starch accumulation
	13 August 2023	13 August 2023	18	Mature harvest
2	5th July 2023	5th July 2023	40	Tuber formation
	18 July 2023	18 July 2023	40	Tuber expansion
	3 August 2023	3 August 2023	40	Starch accumulation
	14 August 2023	14 August 2023	40	Mature harvest
3	6 July 2023	6 July 2023	36	Tuber formation
	20 July 2023	20 July 2023	36	Tuber expansion
	5 August 2023	5th August 2023	36	Starch accumulation
	18 August 2023	18 August 2023	36	Mature harvest

**Table 6:** MicaSense RedEdge-P sensor spectral band information and sensor specifications.

Spectral Band	Center Wavelength/nm	Bandwidth/nm	Pixel Resolution	Field of View
Blue (Band-1)	475	32	1456 × 1088 (1.6 MP)	50°HFOV × 38°VFOV
Green (Band-2)	560	27	1456 × 1088 (1.6 MP)	50°HFOV × 38°VFOV
Red (Band-4)	668	16	1456 × 1088 (1.6 MP)	50°HFOV × 38°VFOV
NIR (Band-5)	717	12	1456 × 1088 (1.6 MP)	50°HFOV × 38°VFOV
Red edge (Band-6)	842	57	1456 × 1088 (1.6 MP)	50°HFOV × 38°VFOV
Panchromatic (Band-3)	634.5	463	2464 × 2056 (1.6 MP)	44°HFOV × 38°VFOV

**Table 7:** List of commonly used vegetation indices relating to physiology and canopy structure.

Abbreviation	Full Name	Formulas	Reference
GRVI	Green ratio vegetation index	NIR/G	[50]
MCARI	Modified chlorophyll absorption in reflectance index	((RE − R) − 0.2 × (RE − G)) × (RE/R)	[51]
MCARI2	Modified chlorophyll absorption in reflectance index 2	1.52 × (NIR − R) − 1.3 × *(NIR − G)/((2 × NIR + 1)^2^ − (6 × NIR − 5 × (R)^0.5^) − 0.5)^0.5^	[52]
NDRE	Normalized difference red edge index	(NIR − RE)/(NIR + RE)	[50]
NDVI	Normalized difference vegetation index	(NIR − R)/(NIR + R)	[11]
RDVI	Renormalized difference vegetation index	(NIR − R)/(NIR + R)^0.5^	[13]
SR	Simple ratio index	NIR/R	[12]
TCARI	Transformed chlorophyll absorption ratio	3 × ((RE − R) − 0.2 × (RE − G)*(RE/R))	[53]
WDRVI	Wide dynamic range vegetation index	(0.1 × NIR − R)/(0.1 × NIR + R)	[53]
NDI	Difference vegetation index	(NIR − RE)/(NIE + R)	[54]
MSR	Modified simple ratio index	(NIR/R − 1)/((NIR/R)^0.5^ + 1)	[55]
GCI	Green chlorophyll index	NIR/G − 1	[56]
RECI	Red-edge chlorophyll index	NIR/RE − 1	[56]
TDVI	Transformed difference vegetation index	(0.5 + (NIR − R)/(NIR + R))^2^	[57]

**Table 8:** Growth process ratio (GPR) of experimental cultivars at different times.

Growth Phase	Z35	Z5	Z27	Z49	Z19
S1	0.28	0.27	0.20	0.19	0.17
S2	0.47	0.44	0.34	0.31	0.29
S3	0.68	0.65	0.51	0.47	0.44
S4	0.85	0.80	0.62	0.57	0.54

## Data Availability

No new data were created or analyzed in this study. Data sharing is not applicable to this article.
